# Utility-preserving anonymization for health data publishing

**DOI:** 10.1186/s12911-017-0499-0

**Published:** 2017-07-11

**Authors:** Hyukki Lee, Soohyung Kim, Jong Wook Kim, Yon Dohn Chung

**Affiliations:** 1 Department of Computer Science and Engineering, Korea University, 145 Anam-ro, Seongbuk-gu, Seoul, 02841 Republic of Korea; 2 Department of IT Convegence, Korea University, Seoul, 145 Anam-ro, Seongbuk-gu, 02841 Republic of Korea; 3 Department of Media Software, Seoul, 20-Gil, Hongji-dong, Seongbuk-gu, 03016 Republic of Korea

**Keywords:** Medical privacy, Data anonymization, Utility-preserving data publishing, K-anonymity

## Abstract

**Background:**

Publishing raw electronic health records (EHRs) may be considered as a breach of the privacy of individuals because they usually contain sensitive information. A common practice for the privacy-preserving data publishing is to anonymize the data before publishing, and thus satisfy privacy models such as *k*-anonymity. Among various anonymization techniques, generalization is the most commonly used in medical/health data processing. Generalization inevitably causes information loss, and thus, various methods have been proposed to reduce information loss. However, existing generalization-based data anonymization methods cannot avoid excessive information loss and preserve data utility.

**Methods:**

We propose a utility-preserving anonymization for privacy preserving data publishing (PPDP). To preserve data utility, the proposed method comprises three parts: (1) utility-preserving model, (2) counterfeit record insertion, (3) catalog of the counterfeit records. We also propose an anonymization algorithm using the proposed method. Our anonymization algorithm applies full-domain generalization algorithm. We evaluate our method in comparison with existence method on two aspects, information loss measured through various quality metrics and error rate of analysis result.

**Results:**

With all different types of quality metrics, our proposed method show the lower information loss than the existing method. In the real-world EHRs analysis, analysis results show small portion of error between the anonymized data through the proposed method and original data.

**Conclusions:**

We propose a new utility-preserving anonymization method and an anonymization algorithm using the proposed method. Through experiments on various datasets, we show that the utility of EHRs anonymized by the proposed method is significantly better than those anonymized by previous approaches.

## Background

### Motivation

In recent years, various health and medical institutions have collected a large amount of medical data, called Electronic Health Records (EHRs). These data are valuable resources that can be used for the prevention of disease, medical decision making, and many other areas of healthcare. Furthermore, various medical data besides EHRs are also widely used in the health domain [1, 2]. Accordingly, data owners have attempted to use the data gathered to make profits through publishing or outsourcing of the data to research organizations.

However, the EHR data usually contain sensitive information such as diagnosis and medication. If data subjects pertaining to sensitive information are disclosed to others, privacy can be breached. For this reason, many countries protect individuals’ privacy in data publishing by laws. These laws allow the publication of only privacy-preserved data. For example, the US Health Insurance Portability and Accountability Act (HIPAA) privacy rule grants the publication of medical information for public purposes without a patient’s consent, if the privacy is preserved adequately. In European Union, privacy regulations are more strict. If data privacy is breached, the data have to be erased [3]. Data anonymization can preserve privacy by eliminating identifiability from the dataset, i.e., the link between sensitive information and people. However, removing Personally Identifiable Information (PII) is not sufficient for eliminating identifiability. A combination of characteristic information (e.g., sex, zipcode, and age), called quasi-identifiers, can play the role of an identifier [4]. To prevent the breach of privacy by quasi-identifiers, *k*-anonymity was proposed [4]. *k*-anonymity means each record contained in a released dataset cannot be distinguished from at least *k*-1 other individuals. *k*-anonymity is used as the privacy criteria in real applications such as the ‘Family Educational Rights and Privacy Act’ (FERPA) [5] of US and the ‘Guidelines for De-identification of Personal Data’ of South Korea [6].

A typical method to achieve *k*-anonymity is generalization. Generalization involves transforming values of quasi-identifiers into more general values to make a person indiscernible from several people. For example, suppose that we release a 4-anonymous dataset for the raw EHR dataset in Table 1, in which *name* is a direct identifier, *age, sex, and zipcode* are quasi-identifiers, and *disease* is a sensitive attribute. The identifier is removed, and the quasi-identifiers are generalized to anonymize the data; the sensitive information should not be removed or modified because it is a critical attribute for analysis. The anonymized result is shown in Table 2. Each record is generalized to an indistinguishable group, called the *equivalent class*, <[35-66], *, [22071-55324] >. As a result of generalization, each record is related to at least three identical records, and thus, the dataset in Table 2 satisfies the 4-anonymity.

**Table 1:** Original EHR data

Name	Age	Sex	Zipcode	Disease
Mary	37	F	22071	Pneumonia
Alice	35	F	22098	Diabetes
Betsy	36	F	23061	Anemia
David	61	M	55107	Pneumonia
Tom	63	M	55099	Diabetes
James	66	M	55324	Diabetes
Eric	62	M	55229	Pneumonia

**Table 2:** 4-anonymous version of Table 1

Age	Sex	Zipcode	Disease
[35−66]	*	[22071−55324]	Pneumonia
[35−66]	*	[22071−55324]	Diabetes
[35−66]	*	[22071−55324]	Anemia
[35−66]	*	[22071−55324]	Pneumonia
[35−66]	*	[22071−55324]	Diabetes
[35−66]	*	[22071−55324]	Diabetes
[35−66]	*	[22071−55324]	Pneumonia

Note that anonymization achieved with generalization-only approaches inevitably distort the records. Therefore, over-generalization negatively affects the analysis of the anonymized dataset. For example, when performing the age-period-cohort analysis using Table 2, as the *age* is over-generalized, it is difficult to obtain meaningful results. In addition, it is important to prevent over-generalization, as the “doctor in the loop” paradigm increases, which the expert knowledge of the doctor is incorporated into “intelligent” systems (e.g., using interactive machine learning) and enriched with additional information and expert know-how [7].

However, anonymization with generalization-only approaches cannot prevent over-generalization, because there is no other way to organize an equivalent class that consists of dissimilar records for achieving *k*-anonymity. To reduce over-generalization, several approaches were proposed [8–10]. Nevertheless, these methods still cannot avoid over-generalization for all records. Therefore, we propose utility-preserving anonymization to achieve *k*-anonymity with high utility. In this method, we propose a utility-preserving model to guarantee the prevention of over-generalization. Furthermore, we propose an anonymization method that satisfies the proposed model and preserves privacy as well.

### Generalization, suppression, and relocation

**Fig. 1:**
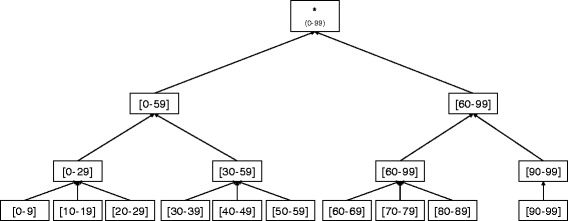
Taxonomy tree of *Age* attribute

In order to generalize the records, numerical attribute values are transformed into range values, and categorical attribute values are transformed into superordinate values. Taxonomy trees are usually employed to describe hierarchies of categorical attributes. Figure 1 shows the taxonomy tree of *Age* attribute. The degree of generalization is quantified as a number between 0 (i.e., minimum generalization) and 1 (i.e., maximum generalization). The degrees of generalization for a numerical attribute *a*_*num*_ and a categorical attribute *a*_*cat*_ are computed as follows.

1 $$ D_{A}(a_{num})=\frac{U_{q}-L_{q}}{U-L}  $$

2 $$ D_{A}(a_{cat})=\frac{|M_{p}|-1}{|M|-1}  $$

*U*_*q*_ and *L*_*q*_ denote the upper bound and lower bound of the generalized interval, respectively. *U* and *L* denote the maximum and minimum value of the whole domain, respectively. *M* denotes the set of leaf nodes in the taxonomy tree and *M*_*p*_ denotes the set of leaf nodes of the subtree rooted by the generalized node. The degree of generalization for a record is defined as the average value of each quasi-identifier attribute’s generalization degree. The degree of generalization for the record *r* is

3 $$ D(r)=\frac{1}{n} \sum\limits_{i=1}^{n} D_{A}(a_{i})  $$

where *n* is the number of attributes. For instance, in the 4-anonymous Table 2, *Age* is a numerical attribute and its domain is [0-99]. Generalization degree for *Age* of the first record is (66−35)/(99−0)=0.31. The other attributes’ generalization degrees are (2−1)/(2−1)=1 and (33254−1)/(100000−1)=0.33, respectively. Therefore, the generalization degree of the first record is (0.31+1+0.33)/3=0.55.

There are two types of anonymization methods that are used in conjunction with generalization for reducing over-generalization: suppression and relocation [8, 9]. Suppression involves the removal of outliers, and relocation involves the changing of the quasi-identifiers of outliers. The presence of outliers is one of the main causes of over-generalization. This is because the outliers are distant from other records, as well as the number of them is not sufficient that they can organize equivalent classes by themselves. In these methods, the values of outliers are removed or changed. Therefore, they can avoid organizing an equivalent class that collects the outlier with other records, and can less generalize a majority of records. Table 3 is obtained from Table 1 by suppressing the first three records. On the other hands, Table 4 is acquired by relocating the first three records in Table 1. Quasi-identifiers of the first three records are relocated to the quasi-identifiers of the other records. By the suppression and relocation of outlier records, the remaining records are not over-generalized.

**Table 3:** 4-anonymous version of Table 1 with suppression

Age	Sex	Zipcode	Disease
*	*	*	*
*	*	*	*
*	*	*	*
[61−66]	M	[55099−55324]	Pneumonia
[61−66]	M	[55099−55324]	Diabetes
[61−66]	M	[55099−55324]	Diabetes
[61−66]	M	[55099−55324]	Pneumonia

**Table 4:** 4-anonymous version of Table 1 with relocation

Age	Sex	Zipcode	Disease
[61−66]	M	[55099−55324]	Pneumonia
[61−66]	M	[55099−55324]	Diabetes
[61−66]	M	[55099−55324]	Anemia
[61−66]	M	[55099−55324]	Pneumonia
[61−66]	M	[55099−55324]	Diabetes
[61−66]	M	[55099−55324]	Diabetes
[61−66]	M	[55099−55324]	Pneumonia

### Data utility

The main objective of *k*-anonymization is privacy protection; however, it is also important that the anonymized dataset should be as useful as possible. There are various *k*-anonymization of a given dataset, but one having the highest utility is desirable. In PPDP, a data owner does not know how the published data will be analyzed by recipients, thus the data utility is measured by the quality of the anonymized dataset. We mainly focus on information loss and data truthfulness for assessing data utility, because these can cover the entire quality of the anonymized dataset in the proposed method (the details will be described later). Information loss refers to the amount of loss caused by generalization. Data truthfulness implies that each anonymized record corresponds to a single original record [11]. Relocated records cannot correspond to original records; thus, they are untruthful. In privacy-preserving data publishing, it is important that a published dataset is truthful. If a published dataset is not truthful, it is difficult to use the results of the data analysis, because false-positive and false-negative results may be obtained. For example, in Table 1, there is a female patient with *Anemia* in her 30s. However, in Table 4, there is no female *Anemia* patient, and in addition, it contains a male *Anemia* patient in his 60s that does not exist in Table 1.

To quantify data utility, various quality metrics are proposed, such as classification metric discernibility metric (DM) [12], loss metric (LM) [13], and reconstruction error (RCE) [14]. DM measures the cardinality of the equivalent class. DM considers only the number of records in the equivalent class; thus, DM does not capture information loss caused by generalization. LM can measure both the cardinality of the equivalent class and information loss. Although LM is more accurate when measuring information loss, it does not consider the data truthfulness. RCE measures the similarity between the original record and the anonymous record. This metric can reflect both information loss and data truthfulness.

### Limitations of the previous methods

While previous methods such as suppression and relocation could reduce information loss, they have some shortcomings that we will here discuss. First, the number of records that can be relocated or suppressed is limited. Suppression and relocation harm data truthfulness. Therefore, data suppression and relocation are only performed on negligible amounts of records for preserving data truthfulness. If the number of outliers exceeds the limitation, over-generalization cannot be prevented, which leads to unacceptable information loss. For example, in Table 4, there are three relocated records. However, the relocation method cannot be applied, if the number of relocatable records is limited to less than 40% of the total number of records. Second, the quality metric they employed does not measure data truthfulness [9]. In hybrid *k*-anonymity, an LM is used. LM measures information loss caused only by generalization, and not by relocation [13]. For this reason, data utility of anonymized data can be severely decreased despite low LM.

## Methods

### Basic concepts

In this section, we introduce the basic concepts behind the proposed anonymization method. The three main goals of the proposed method are as follows: the anonymized dataset should remain (1) useful, (2) privacy-preserving, and (3) reliable. In other words, the anonymized dataset should not be over-generalized, and it should satisfy the privacy model (*k*-anonymity in this paper) and be truthful. To meet these goals, the proposed method comprises three parts: (1) The first part is to restrict the generalization by using a utility-preserving model, called *h*-ceiling, which implies that the degree of generalization is limited to *h* (Subsection h-ceiling). (2) The second part is to generalize counterfeit records. Both the *k*-anonymity and *h*-ceiling are satisfied by inserting counterfeit records (Subsection Insertion of counterfeit records). (3) The third part is to publish a catalog of the counterfeit records that were inserted in the second part to improve data truthfulness (Subsection Catalog of counterfeit records). The catalog consists of sensitive information of counterfeit records and their number in each group of equivalent class. In addition, we describe a quality metric from the results of the proposed method and propose the implementation of an anonymization algorithm using the proposed method (Subsection Implementation of anonymization algorithm).

### h-ceiling

To prevent over-generalization, we limit the generalization degree to *h*, which is the criterion for over-generalization. We now formally define the concepts of *h*-ceiling.

#### **Definition 1**

(***h*** **-ceiling**) Let OT be an original table and AT be an anonymized table of OT. AT satisfies ***h*** **-ceiling** if and only if *D*(*r*)≤*h*.

For example, Table 5 satisfies 0.02-ceiling because *D*(*r*_1_),…,*D*(*r*_4_) = (2/99+0/1+990/99999)/3 =0.01 and *D*(*r*_5_),…,*D*(*r*_8_) = (6/99+0/1+222/99999)/3 = 0.02.

**Table 5:** 0.02-ceiled and 4-anonymous version of Table 1 with insertion

ClassID	Age	Sex	Zipcode	Disease
1	[35-37]	F	[22071-23061]	Pneumonia
1	[35-37]	F	[22071-23061]	Diabetes
1	[35-37]	F	[22071-23061]	Anemia
1	[35-37]	F	[22071-23061]	Diabetes
2	[61-66]	M	[55099-55324]	Pneumonia
2	[61-66]	M	[55099-55324]	Diabetes
2	[61-66]	M	[55099-55324]	Diabetes
2	[61-66]	M	[55099-55324]	Pneumonia

### Insertion of counterfeit records

We describe the insertion of counterfeit records for achieving *h*-ceiling and *k*-anonymity. The counterfeit records are inserted into equivalent classes that satisfy *h*-ceiling but not *k*-anonymity. The counterfeit records have the same quasi-identifiers as the records of the equivalent class, while the sensitive information is randomly selected within the domain of the sensitive attribute. For example, Table 6 cannot satisfy *k*-anonymity, because there are only three records in class 1. Therefore, the counterfeit record is inserted into class 1 to satisfy 4-anonymity as shown in Table 5. A counterfeit record is defined as follows.

**Table 6:** 0.02-ceiled version of Table 1

ClassID	Age	Sex	Zipcode	Disease
1	[35-37]	F	[22071-23061]	Pneumonia
1	[35-37]	F	[22071-23061]	Diabetes
1	[35-37]	F	[22071-23061]	Anemia
2	[61-66]	M	[55099-55324]	Pneumonia
2	[61-66]	M	[55099-55324]	Diabetes
2	[61-66]	M	[55099-55324]	Diabetes
2	[61-66]	M	[55099-55324]	Pneumonia

#### **Definition 2**

(**Counterfeit record**) We define a record *r*∈ AT is a counterfeit record, if and only if *f* ^−1^(*r*)∉OT, where *f*() be an anonymization function and *f* ^−1^() be an inverse function of *f*()

For example, Table 5 is an anonymized table that satisfies 0.02-ceiling and 4-anonymity by adding one counterfeit record, <[35-37], F, [22071-23061], Diabetes > (fourth record).

### Catalog of counterfeit records

The definition and schema of the catalog of counterfeit records are as follows.

#### **Definition 3**

(Catalog for counterfeit records) Let *E*_*t*_(*t*=1,…,*l*) be an equivalent class in AT. A group of equivalent classes *G*_*i*_(*i*=1,…,*m*) consists of several equivalent classes in *E*_*t*_. The catalog has schema

$$Catalog(ClassID\ list, Sensitive\ value, Count) $$

where *ClassIdList* is a list of equivalent class ids in same *G*_*i*_.

The ID list should be organized considering for privacy during creation of the catalog; else, privacy can be breached by the catalog. For example, some adversaries obtain Table 5, and Table 7 and they try to extract personal information. They can remove a record that has *Diabetes* in class 1. Then, only three indistinguishable records exist in class 1; hence 4-anonymity is breached. Although an adversary has some information on counterfeit records, the adversary cannot eliminate counterfeit records when the counterfeit records are not identified [15]. The following lemma shows when the records cannot be eliminated.

**Table 7:** Privacy breached catalog of counterfeit records for Table 5

IDList	Disease	Count
1	Diabetes	1

#### **Lemma 1**

Let $E^{i}_{t}$ be a certain equivalent class in a group *G*_*i*_ and $\mathbb {E}^{i}_{t}$ be a set of other equivalent classes in *G*_*i*_, except $E^{i}_{t}$. If the sum of none-counterfeit records in $\mathbb {E}^{i}_{t}$ for each sensitive value is more than or equal to the number of counterfeit records in $E^{i}_{t}$, then adversaries cannot identify counterfeit records in $E^{i}_{t}$

#### *Proof*

Let $o^{i}_{t}(s)$ be the sum of none-counterfeit records for a certain sensitive value *s* in $\mathbb {E}^{i}_{t}$, $u^{i}_{t}(s)$ be a sum of counterfeit records for *s* in $\mathbb {E}^{i}_{t}$, and $p^{i}_{t}(s)$ be the number of counterfeit records for *s* in $E^{i}_{t}$. Assume that at least one counterfeit record exists in $E^{i}_{t}$ and that $o^{i}_{t}(s)$ is more than or equal to *p*_*s*_. To ensure that at least one counterfeit record exists in $E^{i}_{t}$, the following inequation should be satisfied by the pigeonhole principle.

$$p^{i}_{t}(s)+u^{i}_{t}(s)>o^{i}_{t}(s)+u^{i}_{t}(s) $$

It is contradictory to the assumption $p^{i}_{t}(s) \leq o^{i}_{t}(s)$. □

The ID list should be organized for preserving privacy by Lemma 1. For example, Table 8 is a well-grouped catalog. Counterfeit *Diabetes* patients can be located in both class 1 and class 2, and thus, adversaries cannot be sure where counterfeit records are located.

**Table 8:** Catalog of counterfeit records for Table 5

IDList	Disease	Count
1, 2	Diabetes	1

Table 5 is the 4-anonymous and 0.02-ceiled version of Table 1, and Table 8 shows the catalog of counterfeit records in Table 5. If Table 1 is generalized to satsify 0.02-ceiling, 4-anonymity cannot be satisfied as shown in Table 6, because there are only three records in class 1. Therefore, one counterfeit record is inserted to satisfy 4-anonymity, as seen in Table 5. The catalog shows that there is one counterfeit *Diabetes* patient record in a equivalent group of class 1 or class 2. If data recipients require a more precise dataset without considering data truthfulness, they can use the anonymized dataset in Table 5 as it is. If the data recipients require a truthful dataset, they can handle the dataset as Table 9 using Table 8. The data recipients must remove the counterfeit record to obtain the truthful dataset. Although they cannot know exactly which equivalent class the counterfeit record belongs to, they can remove the counterfeit record using Table 8. To remove the counterfeit record, the one *Diabetes* record in each equivalent class should be suppressed. After that, the recipients can remove one of the suppressed records because one of the suppressed records is a real record, and one is a counterfeit record. In [15], counterfeit records are inserted to protect the privacy only and there are no other methods to preserve the utility. On the other hand, in the proposed method, counterfeit records are inserted to protect the privacy and preserve the utility. Furthermore, data utility can be preserved through the catalog.

**Table 9:** Truthful version of Table 5

ClassID	Age	Sex	Zipcode	Disease
1	[35-37]	F	[22071-23061]	Pneumonia
1	[35-37]	F	[22071-23061]	Diabetes
1	[35-37]	F	[22071-23061]	Anemia
2	[61-66]	M	[55099-55324]	Pneumonia
2	[61-66]	M	[55099-55324]	Diabetes
2	[61-66]	M	[55099-55324]	Pneumonia
*	*	*	*	Diabetes

### Quality metric

**Fig. 2:**
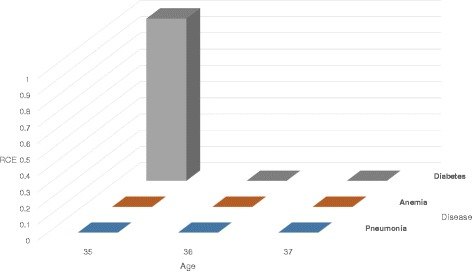
Probability density function of original record for *Age* attribute

**Fig. 3:**
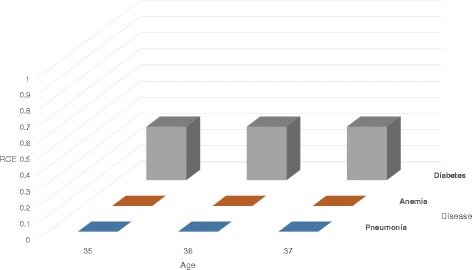
Probability density function of generalized record for *Age* attribute

**Fig. 4:**
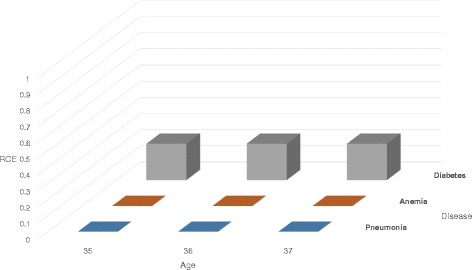
Probability density function of generalized record for *Age* attribute with catalog

In this paper, we adopted two anonymization methods, generalization and insertion of counterfeit records. Therefore, the data utility is determined by the information loss (caused by generalization) and the data truthfulness (caused by insertion). As we mentioned, existing metrics such as DM, LM are not sufficient for measuring quality of the counterfeit records. Because they measure information loss only through quasi-identifier transformation, counterfeit records and catalog cannot be considered in these metrics. For example, for measuring the utility by LM, the most efficient anonymization method is adding *k*-1 counterfeit records to all original records; then LM is always zero. Therefore we will measure utility using RCE. RCE measures utility by using the difference between anonymized data and original data based on the probability density function. For instance, Fig. 2 shows the probability density function of the second record (*Alice*) for *Age* attribute in Table 1. Next, Fig. 3 shows the probability density function of the same record in Table 5. Then, the information loss measured by RCE for *Age* attribute is the difference between Figs. 2 and 3. To calculate RCE of a record, the probability density function is generated for all attributes. The definition of RCE is as follows.

$$ RCE = \sum\limits_{\forall t \in T} \int_{x \in DS} (\tilde{\mathcal{G}}_{t}(x)-\mathcal{G}_{t}(x))^{2} \mathrm{d} x $$

where *DS* is the dimensional space (including all the QI and sensitive attribute) of the record, $\tilde {\mathcal {G}_{t}}$ is the probability density function of the original dataset, and $\mathcal {G}_{t}$ is the probability density function of the anonymized dataset. In the proposed method, the probability density is changed owing to the catalog. In Table 5, there are four *Diabetes* patients in class 1 and class 2, and thus, the probability that the record is counterfeit is 25%. Therefore, the probability density is reduced to 3/4, as in Fig. 4. We measure RCE through the difference between Figs. 2 and 4.

### Implementation of anonymization algorithm

In this section, we present the implementation of the anonymization algorithm using the proposed method. In our methods, we traverse the hierarchical lattice in a bottom-up manner and use heuristics for pruning the node. In addition, we propose a grouping algorithm for catalog publication. The proposed anonymization algorithm applies the full-domain generalization algorithm [16]. The full-domain generalization algorithm finds a solution in the hierarchical lattice that describes all the possible generalization cases as nodes. Many heuristic techniques have been proposed to reduce the search space. Traditional heuristic optimization methods for the algorithm focus on pruning the lattice using the monotonicity of *k*-anonymity [16, 17]. If a node satisfies the *k*-anonymity property, then all the parent nodes linked to that node satisfy *k*-anonymity. On the other hand, if a node does not satisfy the *k*-anonymity property, then all the child nodes linked to that node do not satisfy *k*-anonymity. When using the proposed method, every node satisfies *k*-anonymity, and thus, the lattice cannot be pruned by the monotonicity of *k*-anonymity. Instead, we can prune the lattice by the monotonicity of *h*-ceiling. If a node does not satisfy *h*-ceiling, then all the parent nodes linked to the node cannot satisfy *h*-ceiling, and vice versa. In the proposed method, determining an appropriate value of *h* is important for improving data utility. In our algorithm, if the value of *h* is not determined, the algorithm searches every node in the lattice and finds an optimal value of *h* based on RCE.

Algorithm 1 shows the anonymization algorithm. The algorithm begins with the creation of the hierarchical lattice. Pruning the lattice using monotonicity, we only create a part of the lattice, wherein the generalization boundary of a node is less than *h* (line 1), and search all nodes (lines 3-13). When records are generalized, the number of records in a certain equivalent class can be less than *k*. Then, we add counterfeit records to the equivalent class and store the number of the counterfeit records in TempC (lines 7-13). After generalizing the records based on the generalization rule of the node, TempC is grouped (line 14). The *Grouping* function is described in Section 4.2. After grouping, the algorithm calculates data utility. If the catalog is *null*, the result is also *null*(line 15). In the completion phase, compare the data utility of each node and choose the node that has maximum data utility (lines 16-19).





For grouping a catalog with privacy concerns, we use the heuristic grouping method. Algorithm 2 is the pseudo-code of the grouping algorithm. First, we create a list of the set *S*_*e*_ about equivalent classes that have aggregated data in $\hat {T^{*}}$ and counterfeit records in TempC by sensitive information (line 1). For safe grouping, all equivalent classes should find other equivalent classes that have sufficient none counterfeit records to conceal the counterfeit records by Lemma 1. It is harder to find other equivalent classes when the equivalent class has more counterfeit records. Thus, the list *S*_*e*_ is sorted since the equivalent class that is hard to group takes priority (line 3). After sorting, the algorithm finds equivalent classes grouped into the catalog in descending order (lines 6-23). In this phase, we count matched records to find the most appropriate equivalent class. The *matching* function returns the matched count of the counterfeit records of *S*_*i*_ and the none counterfeit records of *S*_*j*_. If *S*_*i*_ or *S*_*j*_ is already grouped, then the counterfeit records of *S*_*i*_ can match the none counterfeit records of another set *S* in the group (line 12). *matching* function returns *null* for all equivalent classes while *remainCounterfeitRecords* is over 0, the equivalent class *S*_*i*_ cannot be protected by grouping; hence, the algorithm returns null (line 18). If the most appropriate equivalent class is found, the algorithm groups the equivalent classes and counts the unconcealed records and puts that records into *remainCounterfeitRecords* (lines 18-21). If the counterfeit records cannot be concealed at a time, the process is repeated.

## Results and discussions

In this section, we present the experimental evaluation of the proposed method. For the evaluation, we use the Adult dataset from the UCI Machine Learning Repository [18], which is a *de facto standard* dataset for measuring the quality of anonymization algorithms. The Adult dataset has 15 attributes and 32,561 rows. In our experiments, we use eight features, seven quasi-identifiers, and one sensitive attribute for all rows. We used three metrics to assess loss of information: (1) LM, (2) RCE, and (3) query error rate.

In addition, we evaluate the effectiveness of the proposed method in a real world analysis through the NPS dataset from HIRA (Health Insurance Review and Assessment service in Korea) [19]. The NPS (National Patients Sample) dataset consists of EHRs of 3% sampled Korean people in 2011. We analyze 1,375,900 records with 6 attributes: *Age*, *Sex*, *Length* *of* *stay* *in* *hospital*, *Location*, *Surgery* *status*, and *Disease*. We consider the first five attributes to be quasi identifiers and the disease attribute to be a sensitive attribute. In the experiments, in addition to reporting results for the *k*-anonymity with *h*-ceiling method presented in this paper, we also report results for the existing *k*-anonymity algorithm in [16].

### LM

**Fig. 5:**
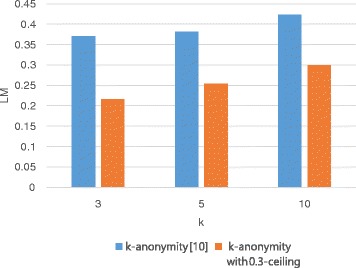
LM variation with k (The lower the bar in the graph, the better are the results)

**Fig. 6:**
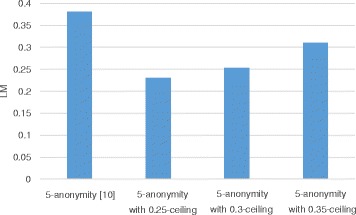
LM variation with h

LM is a general metric for measuring information loss caused by generalization. Figures 5 and 6 show the variation in LM with respect to the privacy parameter *k* and the utility parameter *h*, respectively. In Fig. 5, we compare the *h*-ceiled dataset and the *k*-anonymous datasets when *h* is fixed to 0.3 while the value of *k* varies. The *k*-anonymous datasets have at least 0.37 LM when *k* is 3; LM increases to more than 0.4 when *k* is 10. This is because the data are more generalized to satisfy stricter privacy constraints; that is, LM is proportional to *k*. However, the *h*-ceiled dataset has at most 0.3 LM when *k* is 10 because we constrain the generalization degree by *h*-ceiling. The counterfeit records are inserted with 125, 132, and 103 as the value of *k* increases from 3 to 10. Figure 6 illustrates the LM when *k* is fixed to 5 and *h* varies. The counterfeit records are inserted with 138, 132, and 38 as the value of *h* increases from 0.25 to 0.35. LM increases with *h*. This is because counterfeit records that can decrease LM are inserted to minimize RCE according to the anonymization algorithm. Therefore, LM increases to reduce RCE.

### RCE

**Fig. 7:**
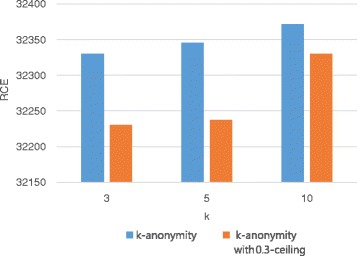
RCE variation with k (The lower the bar in the graph, the better are the results)

**Fig. 8:**
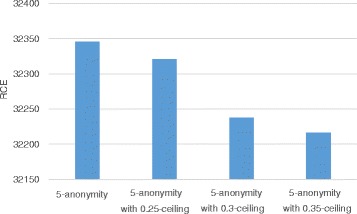
RCE variation with h

In our anonymization method, RCE measures information loss instead of LM, because LM cannot reflect the distortion of counterfeit records. Figures 7 and 8 show the variation in RCE with respect to the privacy parameter *k* and the utility parameter *h*, respectively. Figure 7 shows the comparison between the *h*-ceiled dataset and *k*-anonymous dataset when *h* is fixed to 0.3 and the value of *k* varies. In Fig. 7, *h* is fixed to 0.3 and the value of *k* is varied; the result is compared with k-anonymous data. RCE increases quite a bit when the value of *k* is 10 because a lot of counterfeit records are created to satisfy privacy constraints. Nevertheless, the loss of results in the *h*-ceiled dataset is less than that of results in k-anonymous data. Figure 8 illustrates the variation in RCE with the value of *h*. As the parameter *h* decreases from 0.35 to 0.25, RCE increases. This is because a lot of counterfeit records were created owing to the strict constraint of the generalization degree.

### Query error rate

**Fig. 9:**
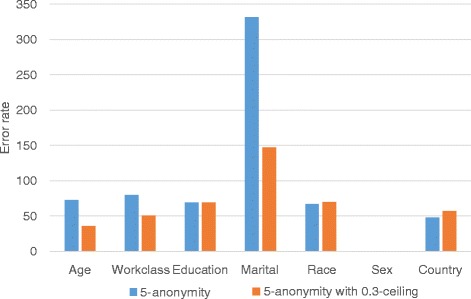
Query error rate

**Fig. 10:**
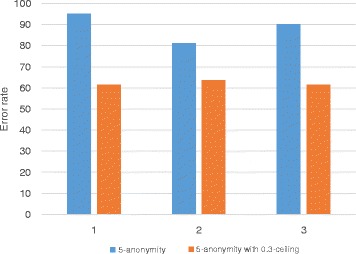
Query error rate variation with number of attributes

We show the result of range queries to verify the effectiveness of the proposed method. We fix the value of *k* as 5 and the value of *h* as 0.3 in these experiments. Figure 9 shows that the error rate of the query result for each attribute. We performed an aggregation query such as SELECT COUNT(*) FROM *Adultdataset* WHERE *Occupation*= ‘*Sales*’ GROUP BY *Age* on both the original dataset and the anonymized dataset, and then compared the results. In *Adult* dataset, the marital status is skewed, and thus, the error rate of the query result on marital status is almost 300%. However, when the proposed method is applied, the error rate can be reduced by half. Further, the error rate on the other attribute for the *h*-ceiled data is also less or nearly equal to the *k*-anonymous data. The error rate on attribute *Sex* is 0 because this attribute is not generalized. Only two values, man and woman, are in this attribute, and thus, the height of the taxonomy tree is 2. This means that if the attribute is generalized, the generalization degree becomes 1 although it is generalized by only one level. In Fig. 10, we show the variation in query error rate with the number of attributes. The error rate on the *h*-ceiled dataset is less than the *k*-anonymous data, regardless of the number of attributes.

### Real world analysis

We present a real-world analysis to illustrate the usefulness of the proposed method. We experimented using the statistical analysis queries used in HIRA [19]. The dataset is anonymized in two ways: (1) *k*-anonymization with *k* = 10, and (2) using the proposed method with *k* = 10 and *h* = 0.2. We further compare the results of the anonymized data with those of the original data by using aggregation queries. The queries are described as follows.

- ***Q***_**1**_: SELECT FLOOR(*Age*/5)*5 AS AgeGroup, COUNT(*) AS No. _*of*_patients FROM *NPS* *dataset* WHERE *Sex*= ‘M’ and *Surgery* *status*= ‘N’ and *Disease*= ‘*stroke*’ GROUP BY FLOOR(*Age*/5)*5
- ***Q***_**2**_: SELECT FLOOR(*Age*/5)*5 AS AgeGroup, COUNT(*) AS No. _*of*_patients FROM *NPS* *dataset* WHERE *Sex*= ‘F’ and *Surgery* *status*= ‘N’ and *Disease*= ‘*stroke*’ GROUP BY FLOOR(*Age*/5)*5
- ***Q***_**3**_: SELECT FLOOR(*Age*/5)*5 AS AgeGroup, AVG(*Length* *of* *stay* *in* *hospital*) AS Average _*length*_*of*_*stay*_*in*_hospital FROM *NPS* *dataset* WHERE *Sex*= ‘M’ and *Surgery* *status*= ‘N’ and *Disease*= ‘*stroke*’ GROUP BY FLOOR(*Age*/5)*5
- ***Q***_**4**_: SELECT FLOOR(*Age*/5)*5 AS AgeGroup, AVG(*Length* *of* *stay* *in* *hospital*) AS Average _*length*_*of*_*stay*_*in*_hospital FROM *NPS* *dataset* WHERE *Sex*= ‘F’ and *Surgery* *status*= ‘N’ and *Disease*= ‘*stroke*’ GROUP BY FLOOR(*Age*/5)*5

**Fig. 11:**
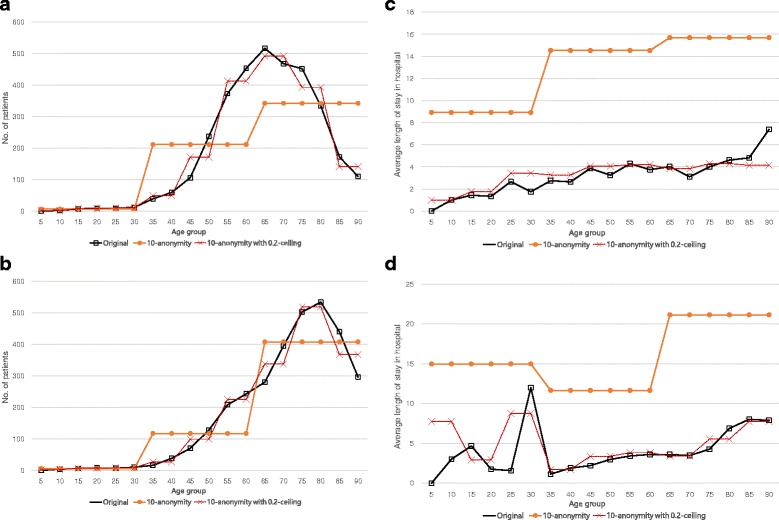
Result of the anaysis queries. **a** Query *Q*_1_ **b** Query *Q*_2_ **c** Query *Q*_3_ **d** Query *Q*_4_

*Q*_1_ and *Q*_2_ represent the number of *stroke* patients for each age group (0-4, 5-9,...,86-90). *Q*_3_ and *Q*_4_ represent the average length of stay in hospital. Figure 11 shows the results of the analysis queries. In Fig. 11-(a) and (b), the x-axis represents the age group (which corresponds to the first projection column of *Q*_1_ and *Q*_2_) and the y-axis represents the number of *stroke* patients (which corresponds to the second projection column of *Q*_1_ and *Q*_2_). In Fig. 11-(c) and (d), the x-axis represents the age group (which corresponds to the first projection column of *Q*_3_ and *Q*_4_) and the y-axis represents the average length of stay in hospital for *stroke* patients (which corresponds to the second projection column of *Q*_3_ and *Q*_4_). In each figure, the result of the *h*-ceiled data is more similar to the original data than that of the *k*-anonymous data. Especially, the proposed method shows better performance in Fig. 11-(c) and (d). Because the average length of stay in hospital is a generalized value, the errors increase as the degree of generalization increases.

## Conclusions

In this paper, we proposed a utility-preserving anonymization method for organizing *h*-ceiled and *k*-anonymous data using two main ideas: counterfeit records, and catalog. Furthermore, we devised an anonymization algorithm with a grouping algorithm and a precise measuring metric of data utility by RCE. Through our experimental results, we demonstrated that *h*-ceiling can prevent the over-generalization and the catalog can preserve data truthfulness of an anonymized dataset.

As possible future works, we can design new anonymization methods that can satisfy *h*-ceiling with other privacy preserving models such as *l*-diversity, *t*-closeness, or MS(*k*, *θ* ^∗^)-anonymity [20–22]. In this paper, we considered only the full-domain generalization which is the most widely used anonymization methodology especially in health/medical domains [16, 17, 23, 24]. As future work, we need extend the proposed method to other types of generalization mechanisms such as clustering and Mondrian [25, 26].

## Abbreviations

DM: Discernibility metric
EHR: Electronic health record
HIPAA: US Health Insurance portability and accountability act
HIRA: Health insurance review and assessment service in Korea
LM: Loss metric
NPS: National patients sample
PPDP: Privacy preserving data publishing
PII: Personally identifiable information
RCE: ReConstruction error

## References

[1] Holzinger A, Dehmer M, Jurisica I (2014). Knowledge discovery and interactive data mining in bioinformatics-state-of-the-art, future challenges and research directions. BMC Bioinforma.

[2] Holzinger A, Jurisica I (2014). Knowledge discovery and data mining in biomedical informatics: The future is in integrative, interactive machine learning solutions. Interactive Knowledge Discovery and Data Mining in Biomedical Informatics.

[3] Malle B, Kieseberg P, Schrittwieser S, Holzinger A (2016). Privacy aware machine learning and the right to be forgotten. ERCIM News.

[4] Sweeney L (2002). k-anonymity: A model for protecting privacy. Int J Uncertain Fuzziness Knowledge-Based Syst..

[5] Family Educational Rights and Privacy Act. 2015. Available at https://ed.gov/policy/gen/guid/fpco/ferpa. 10.1525/jer.2007.2.1.101 19385933

[6] Guidelines for De-identification of Personal Data. 2016. Available at http://privacy.go.kr.

[7] Kieseberg P, Malle B, Frühwirt P, Weippl E, Holzinger A (2016). A tamper-proof audit and control system for the doctor in the loop. Brain Inform.

[8] Sweeney L (2002). Achieving k-anonymity privacy protection using generalization and suppression. Int J Uncertain Fuzziness Knowledge-Based Syst.

[9] Nergiz ME, Gök MZ (2014). Hybrid k-anonymity. Comput Secur.

[10] Prasser F, Kohlmayer F, Kuhn KA (2016). Efficient and effective pruning strategies for health data de-identification. BMC Med Inform Decis Making.

[11] Fung BC, Wang K, Fu AW-C, Philip SY (2010). Introduction to Privacy-preserving Data Publishing: Concepts and Techniques..

[12] Bayardo RJ, Agrawal R (2005). Data privacy through optimal k-anonymization. Proceedings of the 21st International Conference on Data Engineering.

[13] Iyengar VS (2002). Transforming data to satisfy privacy constraints. Proceedings of the Eighth ACM SIGKDD International Conference on Knowledge Discovery and Data Mining.

[14] Xiao X, Tao Y (2006). Anatomy: Simple and effective privacy preservation. Proceedings of the 32nd International Conference on Very Large Data Bases.

[15] Xiao X, Tao Y (2007). M-invariance: Towards privacy preserving re-publication of dynamic datasets. Proceedings of the 2007 ACM SIGMOD International Conference on Management of Data.

[16] Kohlmayer F, Prasser F, Eckert C, Kemper A, Kuhn KA (2012). Flash: Efficient, stable and optimal k-anonymity. Proceedings of the 2012 ASE/IEEE International Conference on Social Computing and 2012 ASE/IEEE International Conference on Privacy, Security, Risk and Trust.

[17] El Emam K, Dankar FK, Issa R, Jonker E, Amyot D, Cogo E, Corriveau JP, Walker M, Chowdhury S, Vaillancourt R (2009). A globally optimal k-anonymity method for the de-identification of health data. J Am Med Inform Assoc.

[18] UCI Repository of Machine Learning Databases. 2013. Available at http://archive.ics.uci.edu/ml.

[19] Health Insurance Review and Assessment Service in Korea. 2012. Available at http://opendata.hira.or.kr.

[20] Machanavajjhala A, Kifer D, Gehrke J, Venkitasubramaniam M (2007). l-diversity: Privacy beyond k-anonymity. ACM Trans Knowl Discov Data (TKDD).

[21] Li N, Li T, Venkatasubramanian S (2007). t-closeness: Privacy beyond k-anonymity and l-diversity. Proceedings of the 21st International Conference on Data Engineering.

[22] Lin WY, Yang DC, Wang JT (2016). Privacy preserving data anonymization of spontaneous ade reporting system dataset. BMC Med Inform Decis Making.

[23] Kohlmayer F, Prasser F, Eckert C, Kemper A, Kuhn KA (2012). Highly efficient optimal k-anonymity for biomedical datasets. Computer-Based Medical Systems (CBMS), 2012 25th International Symposium On.

[24] Kohlmayer F, Prasser F, Kuhn KA (2015). The cost of quality: Implementing generalization and suppression for anonymizing biomedical data with minimal information loss. J Biomed Inform.

[25] Byun JW, Kamra A, Bertino E, Li N (2007). Efficient k-anonymization using clustering techniques. International Conference on Database Systems for Advanced Applications.

[26] LeFevre K, DeWitt DJ, Ramakrishnan R (2006). Mondrian multidimensional k-anonymity. Data Engineering, 2006. ICDE’06. Proceedings of the 22nd International Conference On.

